# HTLV-1 HBZ Viral Protein: A Key Player in HTLV-1 Mediated Diseases

**DOI:** 10.3389/fmicb.2017.02615

**Published:** 2017-12-22

**Authors:** Marco Baratella, Greta Forlani, Roberto S. Accolla

**Affiliations:** Laboratories of General Pathology and Immunology “Giovanna Tosi”, Department of Medicine and Surgery, School of Medicine, University of Insubria, Varese, Italy

**Keywords:** HTLV-1, ATL, HAM/TSP, HBZ, Tax-1

## Abstract

Human T cell leukemia virus type 1 (HTLV-1) is an oncogenic human retrovirus that has infected 10–15 million people worldwide. After a long latency, 3–5% of infected individuals will develop either a severe malignancy of CD4+ T cells, known as Adult T-cell Leukemia (ATL) or a chronic and progressive inflammatory disease of the nervous system designated Tropical Spastic Paraparesis/HTLV-1-Associated Myelopathy (HAM/TSP). The precise mechanism behind HTLV-1 pathogenesis still remains elusive. Two viral regulatory proteins, Tax-1 and HTLV-1 bZIP factor (HBZ) are thought to play a critical role in HTLV-1-associated diseases. Tax-1 is mainly involved in the onset of neoplastic transformation and in elicitation of the host’s inflammatory responses; its expression may be lost during cell clonal proliferation and oncogenesis. Conversely, HBZ remains constantly expressed in all patients with ATL, playing a role in the proliferation and maintenance of leukemic cells. Recent studies have shown that the subcellular distribution of HBZ protein differs in the two pathologies: it is nuclear with a speckled-like pattern in leukemic cells and is cytoplasmic in cells from HAM/TSP patients. Thus, HBZ expression and distribution could be critical in the progression of HTLV-1 infection versus the leukemic state or the inflammatory disease. Here, we reviewed recent findings on the role of HBZ in HTLV-1 related diseases, highlighting the new perspectives open by the possibility of studying the physiologic expression of endogenous protein in primary infected cells.

## Tax-1 and HBZ: The Two Key Players in HTLV-1-Associated Pathologies

Human T-cell leukemia virus type 1 (HTLV-1) was the first oncogenic retrovirus identified in humans (Poiesz et al., 1980; Yoshida et al., 1982). It is currently estimated that HTLV-1 infects at least 10–15 million people worldwide. Large HTLV-1 endemic areas exist in Southern Japan, the Caribbean, Central and South America, the Middle East, Melanesia, and equatorial regions of Africa (Proietti et al., 2005; Gessain and Cassar, 2012). Although most HTLV-1 infected individuals remain asymptomatic carriers (AC) lifelong, about 3–5% of them develop, after many years of clinical latency, a severe malignancy of CD4+ T cells, known as Adult T-cell Leukemia (ATL).

Four clinical subtypes of HTLV-1 associated ATL are described: smoldering, chronic, lymphoma and acute (Shimoyama, 1991). Depending on the subtype and symptoms, the treatment is different and could include: ‘watchful waiting,’ chemotherapy, antiviral therapy, immunotherapy, allogeneic hematopoietic stem cell transplantation (alloHSCT) and targeted therapies (Utsunomiya et al., 2015). However, despite the available treatments, the prognosis is still extremely poor (Nasr et al., 2017). HTLV-1 is also the causative agent of a neurological disease called tropical spastic paraparesis/HTLV-1-associated myelopathy (HAM/TSP) (Gessain et al., 1985), characterized by different neurological features such as spasticity, muscle weakness and sensory deficits. In general, the central function and cranial nerves are strongly compromised and the clinical course is progressive and without remission (Martin et al., 2014). Due to the wide spectrum of symptoms and the inefficiency of the antiviral therapies, the symptomatic treatment represents the current standard of therapy of this disorder.

The pathogenetic mechanisms at the basis of HTLV-1 infection progressing toward ATL or HAM/TSP are not clearly understood although there are sedimented indications that interactions between the viral encoded proteins and a variety of cellular targets are crucial. Two viral proteins, Tax-1 and HTLV-1 bZIP factor (HBZ), encoded by the sense and antisense viral transcripts, respectively, are largely responsible for the malignant transformation and immortalization of the infected T cells. Tax-1 is a potent activator of viral transcription and is involved in neoplastic transformation through modulation of the expression of cellular genes and deregulation of diverse cell signaling pathways involved in cell proliferation, DNA damage repair, and apoptosis (Hall and Fujii, 2005; Matsuoka and Jeang, 2007; Chlichlia and Khazaie, 2010; Tosi et al., 2011; Forlani et al., 2013) The oncogenic properties of Tax-1 are strongly associated to its ability to constitutively activate the nuclear factor kappa B (NF-kB) pathway (Petropoulos et al., 1996; Forlani et al., 2016). It is of note that the alteration of NF-kB signaling pathway could also be involved in the inflammatory state observed in HAM/TSP (Peloponese et al., 2006). Indeed, it has been suggested that pro-inflammatory CD8+ T lymphocytes (CTL), specific for Tax-1-expressing CD4+ T cells, could infiltrate the central nervous system (CNS), kill the target cells and produce inflammatory cytokines, in particular NF-kB-inducible cytokines, that may participate to tissue damage (Asquith and Bangham, 2000). The high immunogenicity of Tax-1 renders it the major target of effectors CTL, and this event may be responsible for the loss of Tax-1 expression during the long clinically latent period leading to overt ATL (Asquith et al., 2005). Genetic and epigenetic modification in the proviral genome are also responsible of Tax-1 silencing (Takeda et al., 2004). Indeed only 40% of ATL patients can express Tax-1 mRNA suggesting that this viral factor is dispensable for the maintenance of leukemia (Matsuoka and Jeang, 2007). Similar data were reported for the inflammatory diseases, in which it was found that 50% of HAM/TSP patiens can express Tax-1 mRNA. (Usui et al., 2008; Saito et al., 2009; Andrade et al., 2013). Interestingly, our recent findings in a restricted sample of patients demonstrated that, at least at protein level, Tax-1 is found in 75% of HAM/TSP cases and in 100% of HTLV-1 AC, but not in ATL cases (Baratella et al., 2017).

At variance with Tax-1, HBZ mRNA (Matsuoka and Green, 2009) and HBZ protein (Raval et al., 2015) are constantly expressed all ATL cases and in HTLV-1 infected individuals, indicating that HBZ is essential not only for cellular transformation but also for the maintenance of leukemic state. HBZ expression increases the proliferation of HTLV-1 infected T cells in culture, and more importantly induces both T-cell lymphomas and systemic inflammation in mice (Satou et al., 2006, 2011). Several studies have suggested a crucial function of HBZ also in HTLV-1-associated inflammatory disorders as it is always found at both mRNA and protein levels in TSP/HAM patients (Andrade et al., 2013). Interestingly, and at variance with Tax-1, most AC do express HBZ mRNA (Saito et al., 2009; Andrade et al., 2013) but not the protein (Baratella et al., 2017). The fact that HBZ protein is scarcely produced in infected cells may explain why is not, or only marginally, discriminated by HBZ specific CTLs in HTLV-1-infected individuals and in HAM/TSP patients (Hilburn et al., 2011; Rowan et al., 2014). The lower immunogenicity of HBZ compared to Tax-1, and the ability of HBZ to inhibit most of Tax-1 activity, could favor the virus immune escape, thus promoting the spreading of infection and the persistence of viral latency. Several studies have indicated a key function of HBZ in supporting and/or maintaining the proliferation of HTLV-1 infected cells and by this, the initiation and persistence of ATL. It has been reported that HBZ promotes ATL cell proliferation by inhibiting apoptosis through different mechanisms: it impairs the binding of AFT3 to p53, thus affecting the activation of p53-mediated apoptosis signaling (Hagiya et al., 2011); it inhibits the transcriptional activation of pro-apoptotic genes as Bim and Fas Ligand, by interfering with FoxO3 (Tanaka-Nakanishi et al., 2014). It was also reported that HBZ impairs anti-viral immunity responses: it binds to NFAT and inhibits the production of Th1 cytokines (particularly IFN-γ); it induces the expression of TIGIT on the cell surface in ATL (Yasuma et al., 2016) and promotes cells migration and proliferation by enhancing CCR4 expression on T-cell surface (Sugata et al., 2016).

Interestingly, several studies have demonstrated that HBZ exerts opposite effects with respect to Tax-1 on signaling pathways. Tax-1 activates while HBZ selectively inhibits the classical NF-κB pathway by affecting the binding of p65-RelA to its consensus DNA sequence and promoting its degradation. This inhibition leads to the activation of the alternative NF-κB pathway (Zhao et al., 2009). It suppresses Tax-1-mediated viral transactivation by interacting with the KIX domain of p300/CBP and impairing the binding of these cellular factors to Tax-1 (Clerc et al., 2008). HBZ suppresses, while Tax-1 activates, Wnt pathway by interacting with the disheveled-associating protein with a high frequency of Leucine residues (DAPLE) (Ma et al., 2013).

Taken together, all these data emphasize the critical role of HBZ in promoting cellular proliferation and the persistence of the viral infection.

## HBZ: Biochemical Aspects and Distinct Subcellular Distribution of Endogenous Protein in ATL and HAM/TSP

Since its discovery in 2002, HBZ has become a crucial hotspot in HTLV-1 research. HBZ gene is encoded by the minus strand of the HTLV-1 RNA genome (Gaudray et al., 2002) and transcribed by a functional promoter, contained in the U5 sequence of the 3′ Long Terminal Repeat (LTR) (Yoshida et al., 2008).

Three major HBZ transcriptional isoforms have been described: the unspliced (usHBZ) form and two alternative spliced form (SP1 and SP2) (Cavanagh et al., 2006; Murata et al., 2006). Regarding the two spliced HBZ isoforms, although both of them have been found in HTLV-1 infected cells, the SP2 variant occurred less frequently than SP1 (Cavanagh et al., 2006). The SP1 spliced and unspliced *HBZ* transcripts are translated into polypeptides of 206 and 209 amino acids, respectively, and they have almost identical sequences except for a stretch of seven amino acids at the N-terminus of the protein (MAAS for SP1 HBZ and MVNFVSA for usHBZ). However, the half-life of usHBZ protein is much shorter than that of SP1 HBZ (Yoshida et al., 2008) and the expression level of SP1 HBZ is four times higher than that of usHBZ in ATL cells (Usui et al., 2008). Nevertheless, it was reported that the two HBZ protein variants exhibit similar functions (Ma et al., 2016), as they were characterized by conserved functional domains: an N-terminal activation domain (AD), a central domain (CD) and a C-terminal basic ZIP domain (bZIP). HBZ contains three nuclear localization signals (NLS) responsible for its nuclear localization (Hivin et al., 2005; Zhao and Matsuoka, 2012) and two functional nuclear export signals (NES) within its N-terminal region (Mukai and Ohshima, 2014). Most of the reported sub-cellular localizations, biochemical interactions and functional aspects related to HBZ have been assessed in cells overexpressing tagged HBZ. Through its bZIP domain, HBZ was reported to interact with CREB/CREB-2, and this association was instrumental to suppress Tax-mediated HTLV-1 viral transcription (Gaudray et al., 2002). Similar experiments have shown that HBZ binds to different proteins of the JUN family via its bZIP domain. Upon binding to HBZ, JunB, and cJun were recruited in nuclear bodies and degraded, thus HBZ reduces the cJun/JunB-mediated transcriptional activation of a series of genes (Basbous et al., 2003; Thébault et al., 2004). Conversely, the association of HBZ to JunD did not inhibit the JunD-mediated transcriptional activation of target genes; indeed HBZ-JunD complex was reported to increase HBZ gene expression (Thébault et al., 2004; Gazon et al., 2012).

Most of the reported HBZ interactions, however, were assessed by artificial overexpressing systems (transfected cells) and thus it has been hard to extrapolate these results to the real situation encountered in infected cells or in leukemic cells from patients. Only recently, the availability of an anti-HBZ monoclonal antibody (mAb), 4D4-F3, isolated in our laboratory, has made it possible to assess endogenous HBZ expression, localization and interaction *in vivo* in HTLV-1 infected and in ATL patient (Raval et al., 2015). Indeed, endogenous HBZ interacts and co-localizes with p300 and JunD. Partial colocalization was observed also for CBP and CREB2 (Raval et al., 2015). By using the 4D4-F3 mAb we were able to quantify the HBZ protein demonstrating that the amount of HBZ in ATL patients is around 0.45 × 10^-2^ pg/cell corresponding to 17.461 molecules/cell, that is 20- to 50-fold lower than the amount expressed in HBZ transfected cells. Similar results were recently obtained by another group (Shiohama et al., 2016). Interestingly, our recent data generated by immunofluorescence with the 4D4-F3 mAb and careful confocal microscopy studies have demonstrated that HBZ protein is expressed in 80 to 100% of ATL cells, in 0.4 to 11% of PBMC of HAM/TSP cells, and very rarely, if any, in PBMC of asymptomatic HTLV-1 carriers (Raval et al., 2015; Baratella et al., 2017).

These studies have also demonstrated that endogenous HBZ protein is localized in the nucleus of ATL cells (Raval et al., 2015; Shiohama et al., 2016) with a similar speckle-like distribution as the one observed in cells transfected with tagged HBZ protein (Gaudray et al., 2002). However, the HBZ nuclear aggregates found in cells overexpressing tagged-HBZ, in particular GFP-HBZ, were shown to be artifacts of chimeric proteins. Although the composition of these nuclear structures containing HBZ is still unknown it has been demonstrated that HBZ-specific nuclear bodies did not overlap with Promyelocytic Leukemia (PML) nuclear bodies (Raval et al., 2015), indicating that HBZ did not co-localize with PML protein. Of note, PML bodies were shown to act as co-activators of Tax-1, without binding to or co-localizing with the viral transactivator (Ariumi et al., 2003)

**FIGURE 1:**
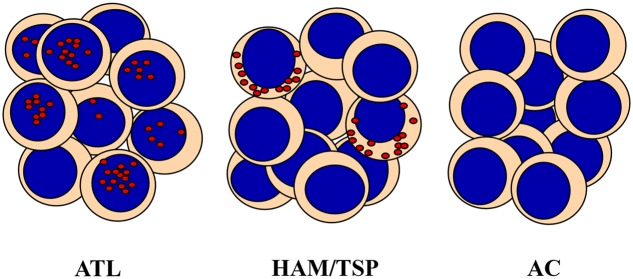
HTLV-1 bZIP factor (HBZ) distribution in PBMC from Adult T-cell Leukemia (ATL), HAM/TSP and asymptomatic carriers (AC). HBZ protein localizes in distinct subcellular compartments and with different percentage in leukemic cells of ATL patients and PBMC of HAM/TSP patients. HBZ is expressed in the nucleus of 90% and in the cytoplasm of 20% of cells isolated from ATL or HAM/TSP patients, respectively. AC do not express detectable levels of HBZ.

One of the most important finding of our studies was the demonstration of the distinct subcellular localization of HBZ protein in HAM/TSP as compared to ATL cells. Until now, HBZ has been always assumed to have an exclusive nuclear localization. Thus, it was relatively unexpected to find that HBZ is exclusively localized in the cytoplasm of PBMC of HAM/TSP patients (**Figure 1**). This cytoplasmic localization was not affected by the presence of leptomycin B, a nuclear export inhibitor, indicating that the viral factor did not shuttle in and out of the nucleus (Baratella et al., 2017). Furthermore, cells expressing cytoplasmic HBZ were almost exclusively found in the CD4+ T cell compartment, very rarely (less than 1%) in CD8+ T cells, and never in B cells or NK cells. Interestingly, the CD4+ HBZ cells, did not expressed the CD25 T cell activation marker (Baratella et al., 2017), thus suggesting that they were either not in rapid proliferation or, if resting, not included in the classical regulatory T cell compartment. This goes in line with recent findings showing that the HBZ-specific humoral immune response correlated with reduced CD4+ T cell activation in HAM/TSP patients (Enose-Akahata et al., 2013). Within this context, the HBZ cytoplasmic localization in HAM/TSP patients may not be appropriate to the generation of peptides that can efficiently bind MHC class I molecules for presentation to, and scrutiny by CTLs. Of further interest was the finding that, at least in the patients’ sample analyzed in our studies, the expression of HBZ and Tax-1 was mutually exclusive (Baratella et al., 2017).

Taken together, these observations let us to propose the cytoplasmic localization of HBZ protein in PBMC of HAM/TSP patients as the first molecular marker of disease, since until now the only associative parameters with the neurological disease were either clinical correlates or the rather non-specific high proviral load (Nagai et al., 1998). Recently, it has been reported that HBZ-transfected cell lines, including the T cell line Jurkat, may partially segregate HBZ in the cytoplasm as result of interaction with THEMIS (Kinosada et al., 2017). With the limitations expressed above on the physiological correlates of HBZ in overexpressing systems, THEMIS may represent a potential HBZ interactor contributing to the cytoplasmic segregation of HBZ also in HAM/TSP patients’ cells. Future studies in patient settings should clarify this point.

## Concluding Remarks and Future Perspectives: The Growing Relevance of HBZ in the Onset of HTLV-1 Associated Diseases

Although several studies have been focussed at clarifying the role of HBZ in HTLV-1-associated diseases, much research is still required to clearly define the molecular and cellular basis of the distinct outcome of viral infection, whether evolving versus the leukemia/lymphoma or versus the neuroinflammatory disorders. In this review, we have summarized the basic molecular aspects of HBZ expression and, importantly, the new findings related to the distinct subcellular localization of HBZ in HTLV-1 infected individuals, AC, affected by ATL or by HAM/TSP neurological disease.

**FIGURE 2:**
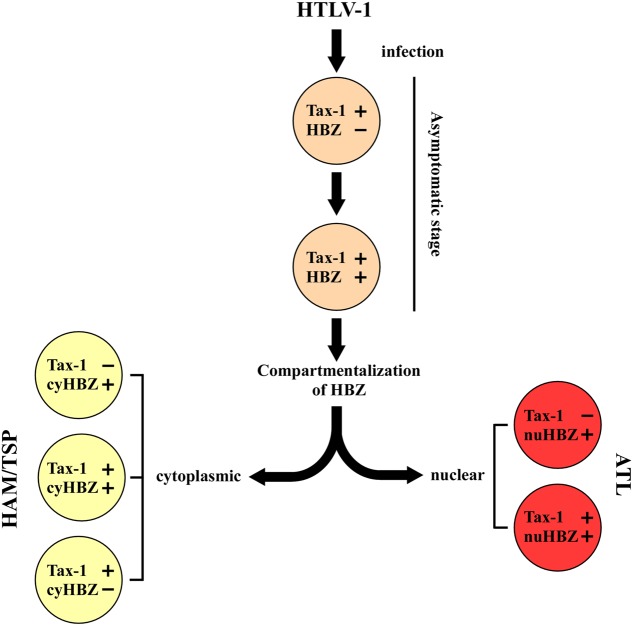
A hypothetical model of disease progression in HTLV-1 infected people. The teory is based on the expression and localization of Tax-1 and HBZ proteins at the single cell level. Primary HTLV-1 infection is associated to the expression of Tax-1. The progression of the disease leads to a different stage characterized by the concominant expression of Tax-1 and HBZ. The subsequent localization of HBZ in the nucleus (nuHBZ) or cytoplasm (cyHBZ) points the disease versus the leukemic or the inflammatory state, as more detailed in the text.

A new paradigm is emerging that suggests a possible roadmap in the evolution of infection based on these new findings (**Figure 2**). We may think that primary HTLV-1 infection is characterized by the expression of Tax-1 (this stage may be observed in PBMC of still asymptomatic patients), followed by the possible transient coexpression of HBZ (yet to be demonstrated). As we have no specific demonstration of this intermediate step, we do not know whether HBZ protein can localize in the nucleus, cytoplasm or both cellular compartments at these early stages of infection. Immediately after, a compartmentalization of HBZ in the cytoplasm can take place in case of evolution of the infection toward HAM/TSP. At this stage, the evolving pathology can either present a concomitant Tax-1 expression in the same cells (rare), or a mutually exclusive expression of Tax-1 and HBZ in different cells, as we observed in our patients. Conversely, in case of evolution toward the leukemic stage, HBZ will be expressed always and only in the nucleus with or without the coexpression of Tax-1. This hypothetical scheme is useful to test several additional hypotheses and investigate previously unforeseen mechanisms. For example, can we pinpoint a stage in which HBZ is compartmentalized in the cytoplasm still without clinical signs of neurological disease and use this parameter also as a prognostic marker of future development to HAM/TSP? Moreover, what are the molecular mechanisms that drive the distinct nuclear or cytoplasmic localization of the HBZ protein? Are there specific interactors that retain HBZ in the two different cellular compartments? Are these mechanism related to the onset and/or the persistence of the distinct pathologies? These and other fascinating questions’ are now, we believe, experimentally amenable to scrutiny.

## Author Contributions

MB, GF, and RA participated in the conception and design of the review and revised the manuscript. All the authors read, critiqued, and approved the final manuscript.

## Conflict of Interest Statement

The authors declare that the research was conducted in the absence of any commercial or financial relationships that could be construed as a potential conflict of interest.
